# Will We Ever Have Conscious Machines?

**DOI:** 10.3389/fncom.2020.556544

**Published:** 2020-12-22

**Authors:** Patrick Krauss, Andreas Maier

**Affiliations:** ^1^Neuroscience Lab, University Hospital Erlangen, Erlangen, Germany; ^2^Cognitive Computational Neuroscience Group, Chair of Linguistics, Friedrich-Alexander University Erlangen-Nürnberg (FAU), Erlangen, Germany; ^3^Chair of Machine Intelligence, Friedrich-Alexander University Erlangen-Nürnberg (FAU), Erlangen, Germany

**Keywords:** machine consciousness, artificial intelligence, theories of consciousness, deep learning, machine learning, philosophy of mind, global workspace, correlates of consciousness

## Abstract

The question of whether artificial beings or machines could become self-aware or conscious has been a philosophical question for centuries. The main problem is that self-awareness cannot be observed from an outside perspective and the distinction of being really self-aware or merely a clever imitation cannot be answered without access to knowledge about the mechanism's inner workings. We investigate common machine learning approaches with respect to their potential ability to become self-aware. We realize that many important algorithmic steps toward machines with a core consciousness have already been taken.

## 1. Introduction

The question of understanding consciousness is in the focus of philosophers and researchers for more than two millennia. Insights range broadly from “*Ignorabimus”—“We will never know*^1^.” to mechanistic ideas with the aim to construct artificial consciousness following Richard Feynman's famous words “*What I cannot create, I do not understand*^2^.”

The major issue that precludes the analysis of consciousness is its subjectivity. Our mind is able to feel and process our own conscious states. By induction, we are also able to ascribe conscious processing to other human beings. However, once we try to imagine to be another species, as Nagel describes in his seminal work “What is it like to be a Bat?”(Nagel, 1974), we immediately fail to follow such experience consciously.

Another significant issue is that we are not able to determine consciousness by means of behavioral observations as Searle demonstrates in his thought experiment (Searle, 1980). Searle describes a room that we cannot enter. One can pass messages written in Chinese to the room and the room returns messages to the outside world. All the messages and questions passed to the room are answered correctly as a Chinese person would. A first conclusion would be that there is somebody in the “Chinese Room” who speaks Chinese and answers the questions. However, the person in the room could also have simply access to a large dictionary that contains all possible questions and the respective answers. When we are not able to understand how the information is actually processed, we will never be able to determine whether a system is conscious or not. In this article, we want to explore these and different thoughts in literature in order to address the problem of consciousness.

Since there exist three different, largely isolated groups in the scientific community aiming to investigate consciousness, i.e., philosophy, neuroscience, and computer science, here, we try to overcome the mutual gaps between these complementary camps of research, by incorporating arguments from each side, thereby providing a balanced overview of consciousness research. We therefore revisit works in philosophy, neuroscience, artificial intelligence, and machine learning. Following the new paradigm of *cognitive computational neuroscience* (Kriegeskorte and Douglas, 2018), we present how the convergence of these fields could potentially also lead to new insights regarding consciousness.

Since philosophy is the root of all scientific research, and in particular the research on consciousness, it was frequently argued that there is a need for more philosophical thinking in scientific research in order to be able to ask the right questions (Thagard, 2009; Rosen, 2015; Laplane et al., 2019). Therefore, we start with the philosophical perspective, and put a special emphasis on the description of the most important arguments and positions from the philosophy of mind.

## 2. The Philosophical Perspective

More than two thousand years ago, Aristotle was convinced that only humans are endowed with a rational soul. All animals, however, live only with the instincts necessary for survival, like biological automata. Along the same line, in the statement “*Cogito ergo sum*” also Descartes realized being self-aware is reserved for human beings. In his view, this insight is fundamental for any philosophical approach (Descartes, 1990).

Modern philosophy went on to differentiate the problem into an easy and a hard problem. While the “easy problem” is to explain its function, dynamics, and structure, the “hard problem of consciousness” Chalmers (1995) is summarized in the Internet Encyclopedia of Philosophy (Weisberg, 2020) as:

“The hard problem of consciousness is the problem of explaining why any physical state is conscious rather than nonconscious. It is the problem of explaining why there is “something it is like” for a subject in conscious experience, why conscious mental states “light up” and directly appear to the subject.”

In order to avoid confusion some scientists prefer to speak of “conscious experience” or only “experience” instead of consciousness (Chalmers, 1995). As already noted, the key problem of deriving models of conscious events is that they can only be perceived subjectively. As such it is difficult to encode such an experience in a way that it can be recreated by others. This gives rise to the so-called “qualia problem” (Crane, 2012) as we can never be sure, e.g., that the color red consciously looks the same to another person. Extension of this line of thought leads again to Nagel's thought experiment (Nagel, 1974).

According to (Weisberg, 2020), approaches to tackle the problem from a philosophical point of view are very numerous, but none of them can be considered to be exhaustive:

- **Eliminativism** (Rey, 1988) demonstrates that the mind is fully functional without the experience of consciousness. Being non-functional, consciousness can be neglected.
- The view of **strong reductionism** proposes that consciousness can be deconstructed into simpler parts and be explained by functional processes. Such considerations gave rise to the Global Work Space Theory (Newman and Baars, 1993; Baars, 1994; Baars and Newman, 1994) or Integrated Information Theory (Tononi, 2004, 2008) in neuroscience. The main critique of this view, is that any mechanistic solution to consciousness that is not fully understood will only mimic true consciousness, i.e., one could construct something that appears conscious that simply isn't as the Chinese Room argument demonstrates (Searle, 1980).
- **Mysterianism** proposes that the question of consciousness cannot be tackled with scientific methods. Therefore any investigation is in vain and the explanatory gap cannot be closed (Levine, 2001).
- In **Dualism** the problem is tackled as consciousness being metaphysical that is independent of physical substance (Descartes, 1990). Modern versions of Dualism exist, but virtually all of them require to reject that our world can be fully described by physical principles. Recently, Penrose and Hammeroff tried to close this gap using quantum theory (Penrose, 1994; Hameroff and Penrose, 2014). We dedicate a closer description of this view in a later section of this article.
- Assuming that metaphysical world and physical world simply do not interact does not require to reject physics and gives rise to **Epiphenomenalism** (Campbell, 1992).

There are further theories and approaches to address the hard problem of consciousness that we do not want to detail here. To the interested reader, we recommend to study the Internet Encyclopedia of Philosophy (Weisberg, 2020) as further reading into the topic.

In conclusion, we observe that a major disadvantage of exploring the subject of consciousness by philosophical means is that we will never be able to explore the inside of the Chinese Room. Thought alone will not be able to open the black box. Neuroscience, however, offers various approaches to explore the inside by means of measurement, which might be suitable to tackle the problem.

## 3. Consciousness in Neuroscience

In 1924, Hans Berger recorded, for the first time, electrical brain activity using electroencephalography (EEG) (Berger, 1934). This breakthrough enabled the investigation of different mental states by means of electrophysiology, e.g., during perception (Krauss et al., 2018a) or during sleep (Krauss et al., 2018b). The theory of cell assemblies, proposed by Hebb (1949), marked the starting point for the scientific investigation of neural networks as the biological basis for perception, cognition, memory, and action. In 1965, Gazzaniga demonstrated that dissecting the corpus callosum which connects the two brain hemispheres with each other results in a split of consciousness (Gazzaniga et al., 1965; Gazzaniga, 2005). Almost ten years later, Weiskrantz et al. discovered a phenomenon for which the term “blindsight” has been coined: following lesions in the occipital cortex, humans loose the ability to consciously perceive, but are still able to react to visual stimuli (Weiskrantz et al., 1974; Weiskrantz and Warrington, 1975). In 1983, Libet demonstrated that voluntary acts are preceded by electrophysiological readiness potentials that have their maximum at about 550*ms* before the voluntary behavior (Libet et al., 1983). He concluded that the role of conscious processing might not be to initiate a specific voluntary act but rather to select and control volitional outcome (Libet, 1985). In contrast to the above mentioned philosophical tradition from Aristotle to Descartes that consciousness is a phenomenon that is exclusively reserved for humans, in contemporary neuroscience most researchers tend to regard consciousness as a gradual phenomenon, which in principle also occurs in animals (Boly et al., 2013), and several main theories of how consciousness emerges have been proposed so far.

### 3.1. Neural Correlates of Consciousness

Based on Singer's observation that high-frequency oscillatory responses in the feline visual cortex exhibit inter-columnar and inter-hemispheric synchronization which reflects global stimulus properties (Gray et al., 1989; Engel et al., 1991; Singer, 1993) and might therefore be the solution for the so called “binding problem” (Singer and Gray, 1995), Crick and Koch suggested Gamma frequency oscillations to play a key role in the emergence of consciousness (Crick and Koch, 1990). Koch further developed this idea and investigated neural correlates of consciousness in humans (Tononi and Koch, 2008; Koch et al., 2016). He argued that activity in the primary visual cortex, for instance, is necessary but not sufficient for conscious perception, since activity in areas of extrastriate visual cortex correlates more closely with visual perception, and damage to these areas can selectively impair the ability to perceive particular features of stimuli (Rees et al., 2002). Furthermore, he discussed the possibility that the timing or synchronization of neural activity might correlate with awareness, rather than simply the overall level of spiking (Rees et al., 2002). A finding which is supported by recent neuroimaging studies of visual evoked activity in parietal and prefrontal cortex areas (Boly et al., 2017). Based on these findings, Koch and Crick provided a framework for consciousness, where they proposed a coherent scheme to explain the neural activation of visual consciousness as competing cellular clusters (Crick and Koch, 2003). Finally, the concept of neural correlates of consciousness has been further extended to an index of consciousness based on brain complexity (Casarotto et al., 2016), which is independent of sensory processing and behavior (Casali et al., 2013), and might be used to quantify consciousness in comatose patients (Seth et al., 2008). While such approaches, known as *perturbational complexity index* (Casali et al., 2013), are designed to assess dynamical or processing complexity, they are not adequate to measure the underlying connectivity or circuitry.

### 3.2. Consciousness as a Computational Phenomenon

Motivated by the aforementioned findings concerning the neural correlates of consciousness, Tononi introduced the concept of integrated information, which according to his “Integrated Information Theory of Consciousness” plays a key role in the emergence of consciousness (Tononi, 2004, 2008). This theory represents one of two major theories of contemporary research in consciousness. According to this theory, the quality or content of consciousness is identical to the form of the conceptual structure specified by the physical substrates of consciousness, and the quantity or level of consciousness corresponds to its irreducibility, which is defined as integrated information (Tononi et al., 2016).

Tegmark generalized Tononi's framework even further from neural-network-based consciousness to arbitrary quantum systems. He proposed that consciousness can be understood as a state of matter with distinctive information processing abilities, which he calls “perceptronium,” and investigates interesting links to error-correcting codes and condensed matter criticality (Tegmark, 2014, 2015).

Even though, there is large consensus that consciousness can be understood as a computational phenomenon (Cleeremans, 2005; Seth, 2009; Reggia et al., 2016; Grossberg, 2017), there is dissent about which is the appropriate level of granularity of description and modeling (Kriegeskorte and Douglas, 2018). Penrose and Hameroff even proposed that certain features of quantum coherence could explain enigmatic aspects of consciousness, and that consciousness emerges from brain activities linked to fundamental ripples in spacetime geometry. In particular, according to their model of orchestrated objective reduction (Orch OR), they hypothesize that the brain is a kind of quantum computer, performing quantum computations in the microtubeles, which are cylindrical protein lattices of the neurons' cytoskeleton (Penrose, 1994; Hameroff and Penrose, 1996; Hameroff, 2001).

However, Tegmark and Koch argue, that the brain can be understood within a purely neurobiological framework, without invoking any quantum-mechanical properties: quantum computations which seek to exploit the parallelism inherent in entanglement, require that the qubits are well-isolated from the rest of the system, whereas on the other hand, coupling the system to the external world is necessary for the input, the control, and the output of the computations. Due to the wet and warm nature of the brain, all these operations introduce noise into the computation, which causes decoherence of the quantum states, and thus makes quantum computations impossible. Furthermore, they argue that the molecular machines of the nervous system, such as the pre- and post-synaptic receptors, are so large that they can be treated as classical rather than quantum systems, i.e., that there is nothing fundamentally wrong with the current classical approach to neural network simulations (Tegmark, 2000; Koch and Hepp, 2006, 2007).

### 3.3. The Global Workspace Theory

**Figure 1:**
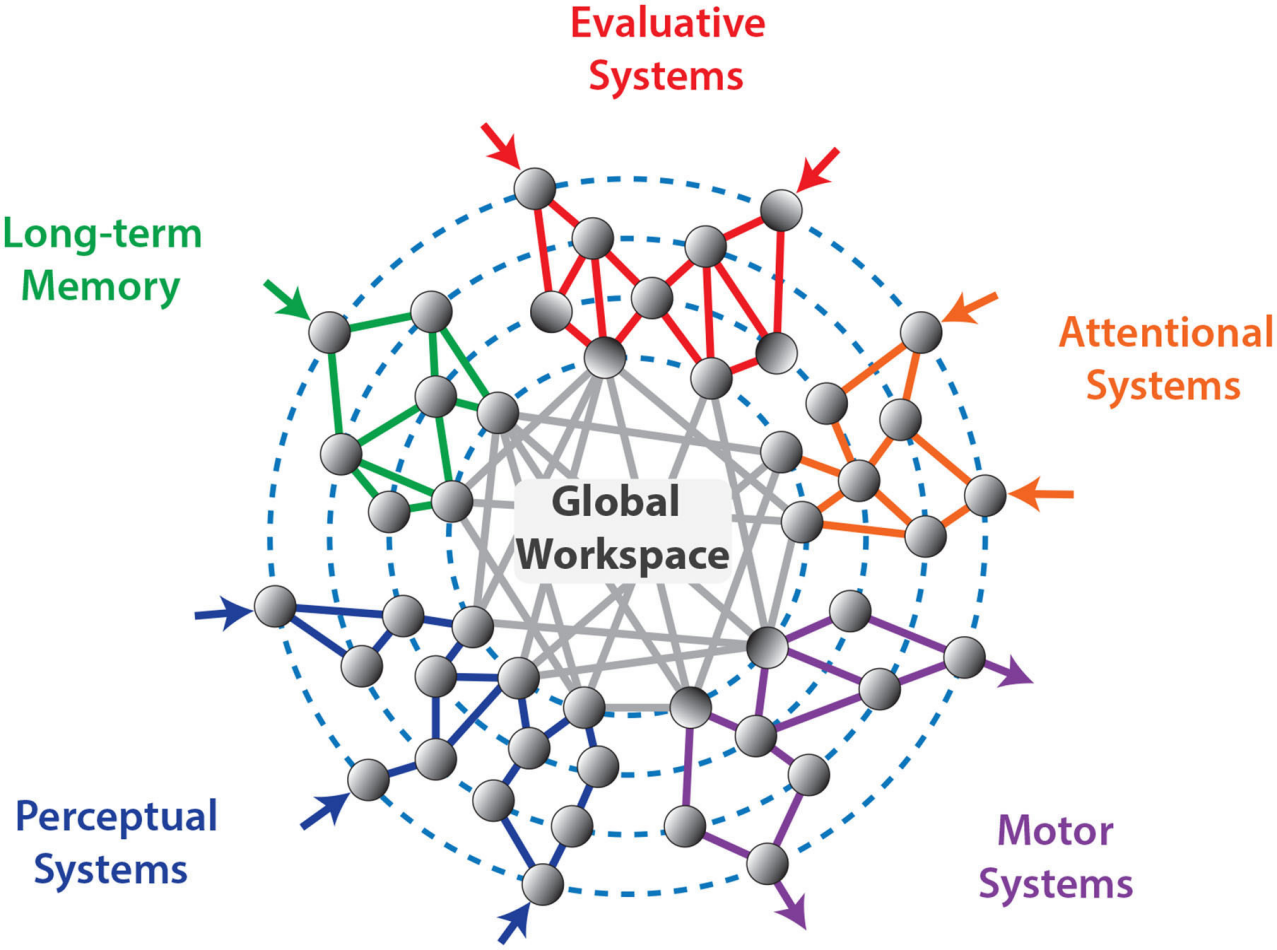
The Global Workspace emerges by connecting different brain areas according to Dehaene.

In the 1990s, Baars introduced the concept of a virtual “Global Workspace” that emerges by connecting different brain areas (Figure 1) to describe consciousness (Newman and Baars, 1993; Baars, 1994, 2007; Baars and Newman, 1994). This idea was taken up and further developed by Dehaene (Dehaene et al., 1998, 2011, 2014; Dehaene and Naccache, 2001; Dehaene and Changeux, 2004; Sergent and Dehaene, 2004). Today, besides the Integrated Information Theory, the Global Workspace Theory represents the second major theory of consciousness, being intensively discussed in the field of cognitive neuroscience. Based on the implications of this theory, i.e., that “*consciousness arises from specific types of information-processing computations, which are physically realized by the hardware of the brain”* (Dehaene et al., 2017), Dehaene argues that a machine endowed with these processing abilities “*would behave as though it were conscious; for instance, it would know that it is seeing something, would express confidence in it, would report it to others, could suffer hallucinations when its monitoring mechanisms break down, and may even experience the same perceptual illusions as humans”* (Dehaene et al., 2017). Indeed, it has been demonstrated recently that artificial neural networks trained on image processing can be subject to the same visual illusions as humans (Gomez-Villa et al., 2018; Watanabe et al., 2018; Benjamin et al., 2019)

### 3.4. Damasio's Model of Consciousness

Damasio's model of consciousness was initially published in his popular science book “The feeling of what happens” (Damasio, 1999). Later Damasio also published the central ideas in peer-reviewed scientific literature (Damasio and Meyer, 2009). With the ideas being published first in a popular science book, most publications on consciousness neglect his contributions. However, we believe that his thoughts deserve more attention. Therefore, we want to introduce his ideas quickly in this section.

The main idea in Damasio's model is to relate consciousness to the ability to identify one's self in the world and to be able to put the self in relation with the world. However, a formal definition is more complex and requires the introduction of several concepts first.

He introduces three levels of conscious processing:

- The fundamental **protoself** does not possess the ability to recognize itself. It is a mere processing chain that reacts to inputs and stimuli like an automaton, completely non-conscious. As such any animal has a protoself according to this definition. However, also more advanced lifeforms including humans exhibit this kind of self.
- A second stage of consciousness is the **core consciousness**. It is able to anticipate reactions in its environment and adapts to them. Furthermore, it is able to recognize itself and its parts in its own image of the world. This enables it to anticipate and to react to the world. However, core consciousness is also volatile and not able to persist for hours to form complex plans.
  In contrast to many philosophical approaches, core consciousness does not require to represent representations of the world in words or language. In fact, Damasio believes that progress in understanding conscious processing has been impeded by dependence on words and language.
- The **extended consciousness** enables human-like interaction with the world. It builds on top of core consciousness and enables further functions such as access to memory in order to create an autobiographic self. Also being able to process words and language falls into the category extended consciousness and can be interpreted as a form of serialization of conscious images and states.

**Figure 2:**
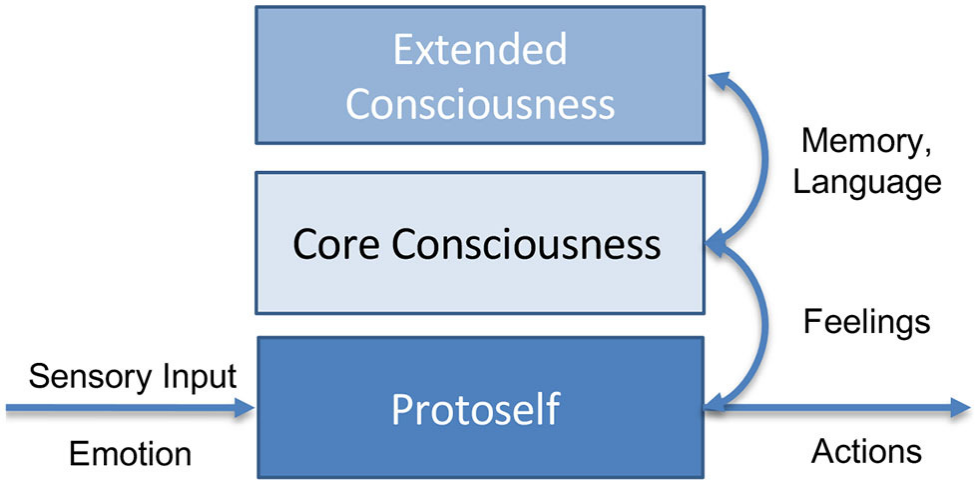
Simplified view of Damasio's model of consciousness: The protoself processes emotions and sensory input unconsciously. Core consciousness arises from the protoself which allows to put the itself into relation. Projections of emotions give rise to higher-order feelings. With access to memory and extended functions such as language processing the extended consciousness emerges.

In Damasio's theory emotions and feelings are fundamental concepts (Damasio, 2001). In particular Damasio differentiates emotions from feelings. **Emotions** are direct signals that indicate a positive or negative state of the (proto-)self. **Feelings** emerge only in conjunction with images of the world and can be interpreted as a second-order emotion that is derived from the world representation and future possible events in the world. Both are crucial for the emergence of consciousness. Figure 2 schematically puts the described terms in relation.

After having defined the above concepts, Damasio now goes on to attempt and describe a model of (core) consciousness. In his theory, consciousness does not merely emerge from the ability to identify oneself in the world or an image of the world. For conscious processing, additionally feeling oneself in the sense of desiring to exist is required. Hence, he postulates a feeling, i.e., a derived second-order emotion, between the protoself and its internal representation of the world. Conscious beings as such want to identify oneself in the world and want to exist. From an evolutionary perspective as he argues, this is possibly a mechanism to enforce self-preservation.

In the context of this article, Damasio's theory is interesting for two major reasons. On the one hand, it describes a biologically plausible model of consciousness, as he assigns all stages of consciousness to certain structures in the brain, and associates them to the respective function. On the other hand, Damasio's model is mechanistic, thus it can be, at least in principle, completely implemented as a computer program.

Summing up, we can conclude that neuroscience is able to describe fundamental processes in the brain that give rise to complex phenomena such as consciousness. However, the different methods of observation in neuroscience are still not sufficient. Neither EEG nor fMRI nor any other contemporary imaging method provide a temporal and spatial resolution that is even close to be enough fine-grained to observe what exactly is happening in the brain *in-vivo* (Maier et al., 2018), i.e., the human brain consisting of about 8.6 × 10^10^ neurons (Herculano-Houzel, 2009) interconnected by approximately 10^15^ synapses (Sporns et al., 2005; Hagmann et al., 2008) is far away from being entirely accessible. At this point, the recent massive progress in artificial intelligence and machine learning, especially deep learning, comes to our attention. In contrast to the human brain, artificial neural networks or any other computational model provide the decisive advantage of being fully accessible at any time, i.e., the state of each parameter can be read out without any restrictions with respect to precision. Furthermore, there is increasing evidence that, even though most artificial neural networks largely lack biological plausibility, they are nevertheless well-suited for modeling brain function. A number of recent studies have shown striking similarities in the processing and representational dynamics between artificial neural networks and the brain (Cichy et al., 2016; Zeman et al., 2020). For instance, in deep neural networks trained on visual object recognition, the spontaneous emergence of number detectors (Nasr et al., 2019), solid shape coding (Srinath et al., 2020), or center-periphery spatial organization (Mohsenzadeh et al., 2020) was observed. Furthermore, grid-like representations known to exist in the entorhinal cortex (Hafting et al., 2005) spontaneously emerge in recurrent neural networks trained to perform spatial localization (Cueva and Wei, 2018) or navigation tasks (Banino et al., 2018).

## 4. Consciousness in Artificial Intelligence

In artificial intelligence (AI) numerous theories of consciousness exist (Sun and Franklin, 2007; Starzyk and Prasad, 2010). Implementations often focus on the Global Work Space Theory with only limited learning capabilities (Franklin and Graesser, 1999), i.e., most of the consciousness is hard-coded and not trainable (Kotov, 2017). An exception is the theory by van Hateren which closely relates consciousness to simultaneous forward and backward processing in the brain (van Hateren, 2019). Yet, algorithms that were investigated so far made use of a global work space and mechanistic hard-coded models of consciousness. Following this line, research on minds and consciousness rather focuses on representation than on actual self-awareness (Tenenbaum et al., 2011). Although representation will be important to create human-like minds and general intelligence (Gershman et al., 2015; Lake et al., 2017; Mao et al., 2019), a key factor to become conscious is the ability to identify a *self* in one's environment (Dehaene et al., 2017). A major drawback of pure mechanistic methods, however, is that the complete knowledge on the model of consciousness is required in order to realize and implement them. As such, in order to develop these models to higher forms such as Damasio's extended consciousness, a complete mechanistic model of the entire brain including all connections is required.

### 4.1. Consciousness in Machine Learning

A possible solution to this problem is machine learning, as it allows to form and train complex models. The topic of consciousness, however, is neglected in the field to a large extent. On the one hand, this is because of the concerns that the brain and consciousness will never be successfully simulated in a computer system (Penrose, 2001; Hameroff and Penrose, 2014). On the other hand, consciousness is considered to be an extremely hard problem and current results in AI are still meager (Brunette et al., 2009).

The state-of-the-art in machine learning instead focuses on supervised and unsupervised learning techniques (Bishop, 2006). Another important research direction is reinforcement learning (Sutton and Barto, 2018) that aims at learning of suitable actions for an agent in a given environment. As consciousness is often regarded to be associated with embodiment, reinforcement learning is likely to be important for modeling of consciousness.

**Figure 3:**
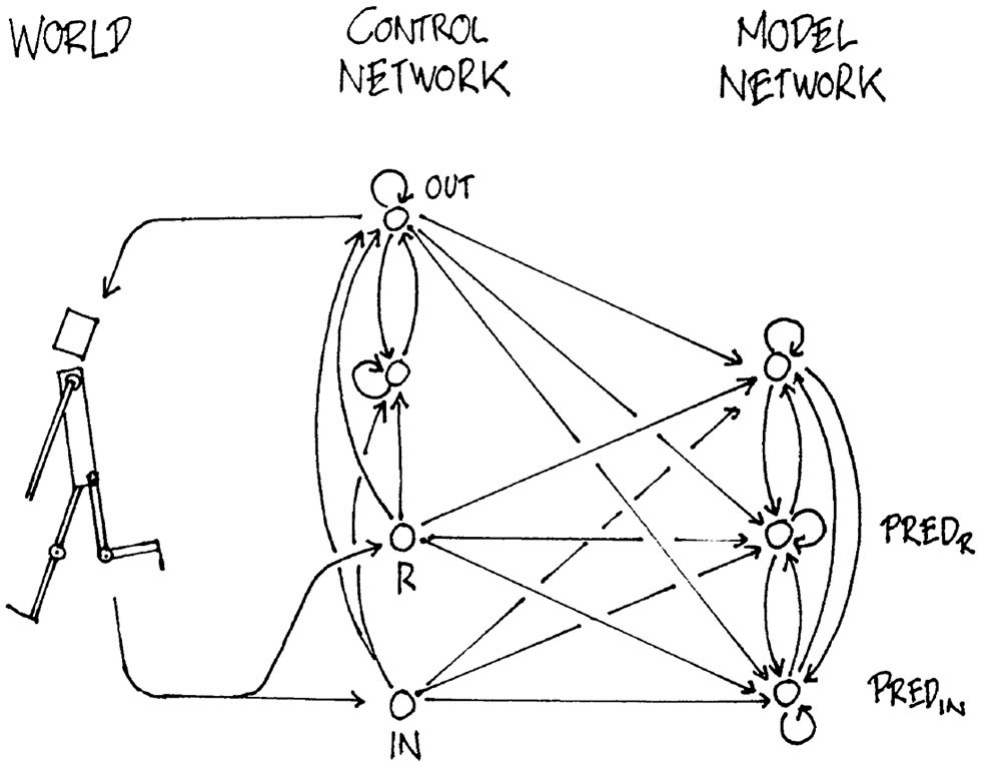
Schmidhuber already proposed a first model for autonomous agents in 1990 (Schmidhuber, 1990). Similar to ideas presented by Damasio, the model receives a reward *R* and input *IN* from the world. The network processes the input, does predictions on the world *PRED*_*IN*_ and predictions about future rewards *PRED*_*R*_. Finally, actions are undertaken in *OUT*. Reprinted with permission.

The earliest work that the authors are aware of attempting to model and create agents that learn their own representation of the world entirely using machine learning date back to the early 1990's. Already in 1990, Schmidhuber proposed a model for dynamic reinforcement learning in reactive environments (Schmidhuber, 1990) and found evidence for self-awareness in 1991 (Schmidhuber, 1991). The model follows the idea of a global work space. In particular, future rewards and inputs are predicted using a world model as shown in Figure 3. Yet, Schmidhuber was missing a theory on how to analyse intelligence and consciousness in this approach. Similar to Tononi (2008), Schmidhuber followed the idea of compressed neural representation. Interestingly, compression is also key to inductive reasoning, i.e., learning from few examples which we typically deem as intelligent behavior.

Solomonoff's *Universal Theory of Inductive Inference* (Solomonoff, 1964) gives a theoretic framework to inductive reasoning. It combines information theory with compression theory and results in a formalization of Occam's razor preferring simple models over complex ones (Maguire et al., 2016), as simple models are more likely from an information theoretic point of view^3^.

Under Schmidhuber's supervision, Hutter applied Solomonoff's theory to machine learning to form a theory of *Universal Artificial Intelligence* (Hutter, 2004). In this theory, intelligent behavior stems from *efficient compression*^4^ of inputs, e.g., from the environment, such that predictions and actions are performed optimally. Again, models capable of describing a global work space play an important role.

Maguire et al. further expand on this concept to extend Solomonoff's and Hutter's theories to also describe consciousness. Following the ideas of Tononi and Koch (Rees et al., 2002) consciousness is understood as data compression, i.e., the optimal integration of information (Maguire et al., 2016). The actual consciousness emerges from binding of information and is inherently complex. As such, consciousness can also not be deconstructed into mechanical sub-components, as the decomposition would destroy the sophisticated data compression. Maguire et al. even provide a mathematical proof to demonstrate that consciousness is either integrated and therefore cannot be decomposed or there is an explicit mechanistic way of modeling and describing consciousness (Maguire et al., 2016).

Based on the extreme success of deep learning (LeCun et al., 2015), also several scientists observed similarities in neuroscience and machine learning. In particular, deep learning allows to build complex models that are hard to analyse and interpret at the benefit of making complex predictions. As such both fields are likely to benefit each other in the ability to understand and interpret complex dynamic systems (Marblestone et al., 2016; Hassabis et al., 2017; Van Gerven, 2017; Kriegeskorte and Douglas, 2018; Barrett et al., 2019; Richards et al., 2019; Savage, 2019). In particular, hard-wiring following biological ideas might help to reduce the search space dramatically (Zador, 2019). This is in line with recent theoretical considerations in machine learning as prior knowledge allows to reduce maximal error bounds (Maier et al., 2019b). Both fields can benefit from these ideas as recent discoveries of e.g., successor representation show (Stachenfeld et al., 2017; Gershman, 2018; Geerts et al., 2019). Several scientists believe that extension of this approach to social, cultural, economic, and political sciences will create even more synergy resulting in the field of *machine behavior* (Rahwan et al., 2019).

## 5. Can Consciousness Emerge in Machine Learning Systems?

After having reviewed philosophy, neuroscience, and the state-of-the-art in AI and machine learning, we can now analyse the most important concepts in the field of machine learning, especially deep learning, to assess whether they have the potential to create consciousness following one of the previous theories. In particular, we focus on the ability of the system to represent a category of self and how this self-awareness is constructed, as all theories of consciousness require at least experiencing the self.

**Figure 4:**
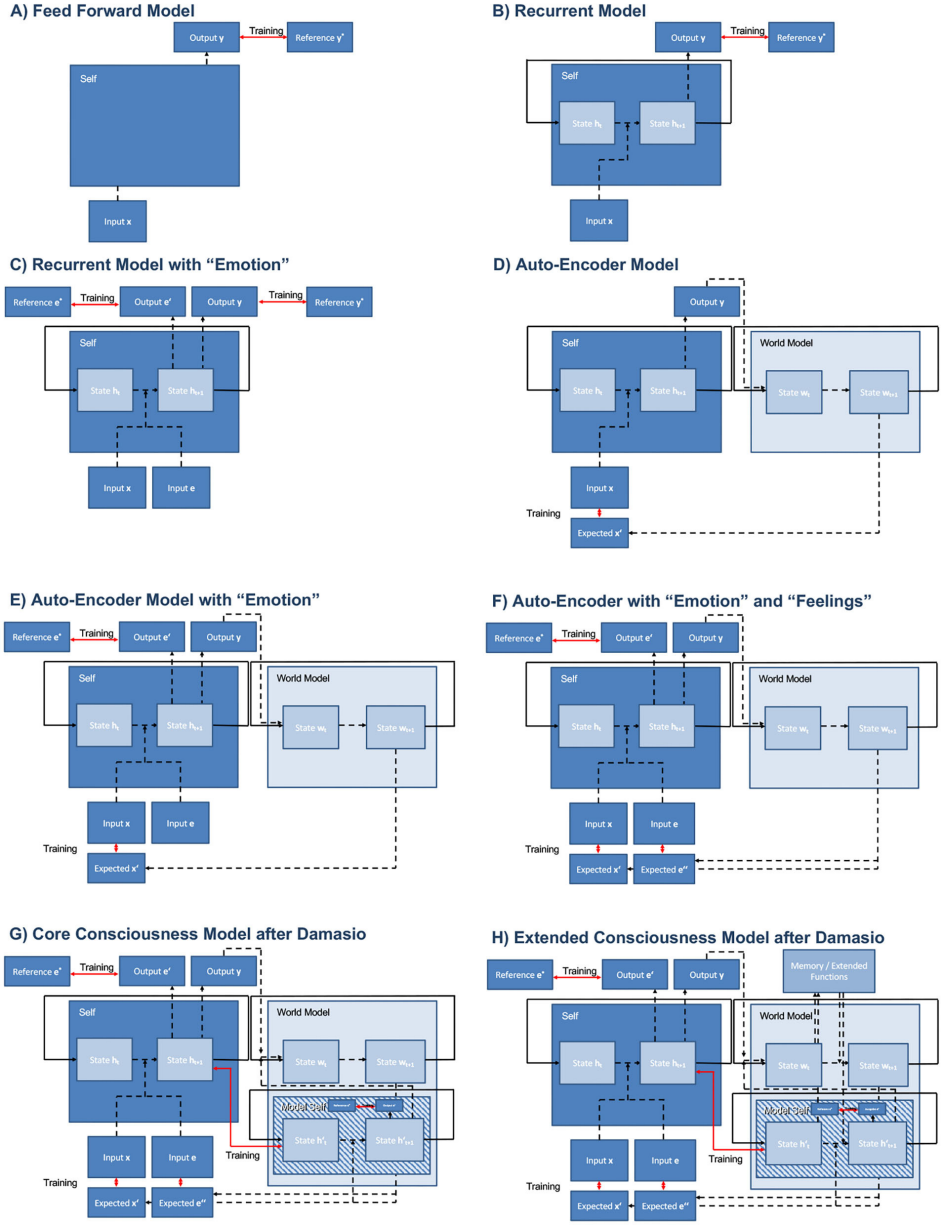
Overview on typical architectures in machine learning and proposed models. Hard-coded paths, i.e., recurrent connections, are indicated by solid black lines, trainable feed-forward connections by dashed black lines, and training losses by red lines. Arrows indicate information flow. While architectures **(A–E)** do not match theories of consciousness, architectures **(F–H)** implement theories by Schmidhuber and Damasio.

In Figure 4, we provide an overview of important models depicted as box-and-arrow schemes, following the standard way to communicate neural network architectures within the machine learning community. We denote *feed-forward connections*^5^ as dashed black lines, *recurrent connections*^6^ as solid black lines, and *training losses*^7^ as red lines. Arrows indicate the direction of information flow.

The trainable feed-forward connections perform certain transformations on the input data yielding the output. In the most simple case, each depicted feed-forward connection could either be realized as a direct link with trainable weights from the source module to the respective target module, a so called perceptron (Minsky and Papert, 2017), or with a single so called hidden layer between source and target module. Those multi-layer neural networks are known to be universal function approximators (Hornik, 1991). Without loss of generality, the depicted feed-forward connections could also be implemented by other deep feed-forward architectures (Maier et al., 2019a) comprising several stacked hidden layers, and could thus be inherently complex^8^.

Figure 4A shows a simple feed-forward architecture which can be trained through supervised learning, i.e., it requires external labeled training data y^*^ to adjust its trainable weights given input *x* to produce output *y*. During training the error between expected output y^*^ and actual output *y* is minimized. Models like this are used in machine learning to classify static input like images for instance (LeCun et al., 2015). In this model, we do not expect the emergence of consciousness.

Figure 4B shows a similar setup, yet with an additional recurrent connect feeding the momentary internal state *h*_*t*_ back to the input. Thus, the subsequent internal state *h*_*t*+1_ and output *y*_*t*+1_ depend not only on the subsequent input *x*_*t*+1_ but also on the previous internal state. Note that we only depict a simple recurrent cell here with time-dependent internal state *h*_*t*_. Without loss of generality, this could also be realized by more complex architectures like gated recurrent units (GRUs) (Cho et al., 2014) or long short-term memory cells (LSTMs) (Hochreiter and Schmidhuber, 1997). Again, models like this fall into the category of supervised learning, i.e., require labeled input data or information about the expected output, respectively. Due to their ability of processing sequential input, these models are widely used in contemporary machine learning, e.g., natural language processing (Young et al., 2018). The emergence of consciousness in those models is neither observed, nor supported by any of the theories presented so far.

In Figure 4C, we introduce the concept of “Emotion” following Damasio's wording. In machine learning terms, this reflects an additional loss. Now, the system receives an additional input *e* that is associated to a valence or value, i.e., a reward. Without loss of generality, we can assume positive entries in *e* to be associated to desirable states for the system and negative values to undesirable states. As such, training using *e* falls into the category of reinforcement learning (Sutton and Barto, 2018) that aims at maximizing future rewards of *e*, by adjusting or choosing appropriate output *y*, i.e., action or behavior. In order to model competing interests and saturation effects, e.g., a full battery does not need to be charged further, we introduce a reference e^*^, i.e., a desired reward, that is able to model such effects. This corresponds to the concept of homeostasis in biology: desired rewards e^*^ correspond to preferred values of metabolic parameters like blood oxygenation level for instance, whereas the actual rewards *e* would correspond to actual values of such metabolic parameters. Like in a feedback loop, the organism seeks to minimize the difference between desired and actual values of metabolic parameters. Note that we deem the system to be able to predict the expected future reward *e*′ from its current state *h*_*t*_ following a so called deep Q-learning paradigm (Mnih et al., 2015). Here we use *e*′ and e^*^ to construct a trainable reinforcement loss, to be able to learn from low-level rewards *e* to adjust the organism's output action or behavior, respectively. In Damasio's model, the totality of all actual low-level rewards *e* correspond to emotions. Although being able to learn, systems like this still need supervision to train the weights producing appropriate output (action / behavior) *y* using a reference. In machine learning, such models fall into the class of model-free reinforcement learning (Sutton and Barto, 2018). Although these models are able to learn playing computer games (Mnih et al., 2015) or board games like Go (Silver et al., 2017) at human level, the emergence of consciousness is not expected, let alone observed, since this setup also does not match any theory of consciousness so far.

As self-awareness is a requirement for base consciousness, we deem a world model, i.e., a model of the organism's environment, to be necessary. Such an approach is shown in Figure 4D, and corresponds to so called auto-encoders which are used in machine learning for dimensionality reduction (Wang et al., 2016) or the construction of compact feature spaces, so called embeddings (Lange and Riedmiller, 2010). Given the organism's produced output (action / behavior) *y*, the world model is used to estimate an expected future input *x*′. In the figure, we chose a recurrent model capturing the sequence of external states of the world *w*_*t*_ that is independent of the sequence of internal states of the agent *h*_*t*_. To gain consciousness, this model misses at least a link from internal to external state and “emotions” that would guide future decisions.

Combining the two previously described models (Figures 4C,D), i.e., adding low-level rewards/emotions to the auto-encoder model results in the model shown in Figure 4E, which corresponds, in contrast to model-free reinforcement learning, to model-based reinforcement learning (Sutton and Barto, 2018). Again world and self are disconnected, hence inhibiting self-representation and self-discovery. Approaches like this are already being explored for video game control (Kaiser et al., 2019).

With a world-model being present, besides predicting future rewards *e*′ from the internal agent state, we are now able to additionally predict future rewards *e*″ that also take into account the external state of the world and the chosen action / behavior *y*. As such Figure 4F is the first one that would implement a trainable version of deep Q-learning. However, development of consciousness is debatable, as the model does not feature a link between the external state of the world *w*_*t*_ and the internal state of the agent *h*_*t*_. If we would add trainable connections from *h*_*t*_ to *w*_*t*_ and vice versa, we would end up with Schmidhuber's Model from 1990 (Schmidhuber, 1990) (Figure 3) for which Schmidhuber found evidence to develop self representation (Schmidhuber, 1991).

Interestingly, Damasio's descriptions follow a similar line in Damasio (1999). We depict a model implementing Damasio's core consciousness in Figure 4G. As Schmidhuber, Damasio requires a connection from the world model *w*_*t*_ to the body control system *h*_*t*_. However, in his view, consciousness does not emerge by itself. It is enforced by a “feeling” that is expressed as a training loss in terms of machine learning. As such, the Damasio model of core consciousness requires a loss that aims at the recovery of the image of the self in the world model. If this is implemented as a loss, we are able to express the desire to exist in the world. If implemented merely as trainable weights, we arrive at the theory of integrated information (Tononi et al., 2016) that creates consciousness as a maximally compressed representation of the world, the self, and their mutual interactions. Interestingly, these considerations also allow the integration of an attention mechanism (Vaswani et al., 2017) and other concepts of resolving context information used in machine learning. Realized in a biological learning framework, e.g., using neuromodulators like dopamine (Russek et al., 2017), the different notions of loss and trainable connections will disappear. Therefore, we hypothesize that from a meta-perspective, the models of Damasio (Damasio, 1999), Schmidhuber (Schmidhuber, 1990), Tononi (Tononi et al., 2016), Koch (Koch et al., 2016), and Dehaene (Dehaene et al., 1998, 2011, 2014; Dehaene and Naccache, 2001; Dehaene and Changeux, 2004; Sergent and Dehaene, 2004) may be basically regarded as different descriptions of the same fundamental principles.

Note that the models of consciousness that we have discussed so far are very basic. They do not take into account higher cognitive functions like language, different kinds of memory (procedural, episodic, semantic), nor any other complex multi-modal forms of processing, e.g., hierarchical action planning, induction, causal inference, or conclusion by analogy. Again, we follow Damasio at this point in Figure 4H in which all of these sophisticated processes are mapped into a single block “Memory / Extended Functions.” Note, although we omit these extended functions, we are able to integrate them using trainable paths. As such, the model of core consciousness (Figure 4G) acts as a kind of“neural operating system” that is able to update and integrate also higher order cognitive functions according to the needs of the environment. We agree with Damasio that this core consciousness is shared by many species, i.e., probably all vertebrates, cephalopods, and perhaps even insects. By increasing the number of “extended functions,” the degree of complexity and “integrated information” rises measurably, as also observed by Casarotto et al. (2016). In mammals, all higher order, i.e., extended, cognitive functions are located in the cerebral cortex. Hence, the growth of cortex size during mammal evolution, corresponds to an increasing number of extended functions.

This brings us back to the original heading of our section: There are clearly theories that enable modeling and implementation of consciousness in the machine. On the one hand, they are mechanistic to the extend that they can be implemented in programming languages and require similar inputs as humans would do. On the other hand, even the simple models in Figure 4 are already arbitrarily complex, as every dashed path in the models could be realized by a deep neural network comprising many different layers. As such also training will be hard. Interestingly, the models follow a bottom-up strategy such that training and development can be performed in analogy to biological development and evolution. The models can be trained and grown to more complex tasks gradually.

## 6. Discussion

Existence of consciousness in the machine is a hot topic of debate. Even with respect to the simple core consciousness, we observe opinions ranging from “generally impossible” (Carter et al., 2018) through “plausible” (Dehaene et al., 2017) to “has already been done” (Schmidhuber, 1991). Obviously, all of the suggested models cannot solve the qualia problem or the general problem on how to demonstrate whether a system is truly conscious. All of the emerging systems could merely be mimicking conscious behavior without being conscious at all (even Figure 4A). Yet as already discussed by Schmidhuber (1991), we would be able to measure correlates of self recognition similar to neural correlates of consciousness in humans (Koch et al., 2016) which could help to understand consciousness in human beings. However, as long as we have not solved how to provide proof of consciousness in human beings, we will also fail to do so in machines as the experience of consciousness is merely subjective.

Koch and Dehaene discussed the theories of global work space and integrated information as being opposed to each other (Carter et al., 2018). In the models found in Figure 4, we see that both concepts require a strong degree of interconnection. As such, we do not see why both concepts are fundamentally opposing. A global work space does not necessarily have to be encoded in decompressed state. Also, Maguire's view of integrated information (Maguire et al., 2016) is not necessarily impossible to implement mechanistically, as we are able to use concepts of deep learning to train highly integrated processing networks. In fact, as observed by neuroscience (Kriegeskorte and Douglas, 2018), both approaches might support each other yielding methods to construct and reproduce biological processes in a modular way. This allows the integration of representation (Gershman et al., 2015) and processing theories (Sun and Franklin, 2007) as long as they can be represented in terms of deep learning compatible operations (Maier et al., 2019b).

In all theories that we touched in this article, the notion of self is fundamental and the emergence of consciousness crucially requires embodiment. Feedback from internal body states is regarded to be the basis of emotions and feelings. Without emotions and feelings, the system cannot be trained and thus cannot adapt to new environments and changes of circumstances. Furthermore, certain additional cognitive functions are crucial to support, together with core consciousness, the emergence of extended consciousness as observed in humans and other non-human primates, as well as some other higher mammals, birds and cephalopods. These cognitive functions comprise, for instance attention, hierarchical action planning, procedural, episodic, and semantic memory.

In the machine learning inspired models, we assume that a disconnection between environment and self would cause a degradation of the system similar to the one that is observed in human beings in complete locked-in state (Kübler and Birbaumer, 2008) or in chronically curarized rats (Birbaumer, 2006). This homeostatsis, i.e., the regulation of body states aimed at maintaining conditions compatible with life, was also deemed important for the design of feeling machines by Man and Damasio (2019). Note that, a slightly different concept of homeostasis has been introduced by Tononi and Cirelli in the context of the sleep homeostasis hypothesis (Tononi and Cirelli, 2003). There, it is assumed that synaptic potentiation is tied to the homeostatic regulation of slow-wave activity.

Similar to the problems identified by Nagel, also the proposed mechanistic machine learning models will not be able to understand “what it is like” to be a bat. However, the notion of train-/learnable programs and connections or adapters might offer a solution to explore this in the future. Analogously, one cannot describe to somebody “what it is like” to play the piano or to snowboard on expert level unless one really acquires the ability. As such also the qualia problem persists in machine consciousness. However, we are able to investigate the actual configuration of the representation in the artificial neural net offering entirely new levels of insight.

In Damasio's theory, consciousness is effectively created by a training loss that causes the system to “want” to be conscious, i.e., “Cogito ergo sum” becomes “Sentio ergo sum.” Comparison between trainable connections after (Schmidhuber, 1990), attention mechanisms (Vaswani et al., 2017), and this approach are within the reach of future machine learning models which will create new evidence for the discussion of integrated information and global work spaces. In fact, Schmidhuber has already taken up the work on combination of his early ideas with modern approaches from deep learning (Schmidhuber, 2015, 2018).

With models for extended consciousness, even the notion of the Homunculus (Kenny, 2016) can be represented by extension of the self with another self pointer. In contrast to common rejection of the Homunculus thought experiment, this recurrent approach can be trained using end-to-end systems comparable to AlphaGo (Silver et al., 2016).

Damasio also presents more interesting and important work that is mostly omitted in this article for brevity. In Damasio (1999), he also relates structural brain damage to functional loss of cognitive and conscious processing. Also the notion of emotion is crucial in a biological sense and is the driving effect of homeostasis. In Man and Damasio (2019), Damasio already pointed out that this concept will be fundamental for self-regulating robotic approaches.

With the ideas of cognitive computational neuroscience (Kriegeskorte and Douglas, 2018) and the approaches detailed above, we will design artificial systems that approach the mechanisms of biological systems in an iterative manner. With the iterations, the artificial systems will increase in complexity and similarity to the biological systems. With respect to artificial systems and machine learning, we are far away from the complexity of biological neural structures. Yet, we can adopt Damasio's strategy of identifying the presence of certain structures and links within these models. This is also the main contribution of our article. It allows us to link AI methods to Damasio's categories. In our analysis, we also observe that none of the machine learning models today could be mapped to the highest (and human-like) kind of extended consciousness.

However, even if we arrive at an artificial system that performs identical computations and reveals identical behavior as the biological system, we will not be able to deem this system as conscious beyond any doubts. The true challenge in being perceived as conscious will be the acceptance by human beings and society. Hence, requirements for conscious machines will comprise the similarity to biological conscious processes, the ability to convince human beings, and even the machine itself. As Alan Turing already proposed in his *imitation game* in 1950 to decide whether a machine is intelligent or even conscious (Turing, 1950), the ascription of such by other humans is a critical factor. For this purpose, Turing's Test has already been extended to also account for embodiment (French, 2012). However, such tests are only necessary, but not sufficient as Gary Marcus pointed out: Rather simple chat bot models are already able to beat Turing's Test in some occasions (Vardi, 2014).

Turing's test is able to test consciousness and intelligence only from an outside point of view. For the perception of consciousness, the internal point of view, however, is even more important. As Turing observed, the only means to quantify consciousness would be to directly compare experiences or memory contents with each other, or at least indirectly through serializations/projections. One way to serialize thoughts, experiences, and memories is language, and the comparison of such with real human serializations is part of the Turing-Test. However, since language provides only a coarse projection of memory and experience, also its analysis is necessarily coarse and may be feigned on purpose. A better path of creating a quantitative measure of consciousness would be to compare digital serializations of memory with respect to the ability to re-create the conscious experience with sufficient precision in the sense of the Nyquist-Shannon Sampling Theorem (Shannon, 1949). However, this path is still far away in the future as it would require storing and loading of digital memories using neural interfaces.

In this line of thought, we can now also relate to analysis of consciousness in biology: For example, there is the so called *mark and mirror test* (Bard et al., 2006) that shows at what age self-representation appears in humans, or whether animals (corvids) seem to possess this ability. Here, the means of serialization of conscious experience is even more limited. Yet, we are able to understand certain basic feelings such as ones related to self-perception in the mirror by analysis of actions. So, one could interpret the mark and mirror test as a very weak version of the Turing test with respect to core consciousness. Another conjecture to assess the putative existence of artificial consciousness in candidate machine learning systems would be to apply the concept of the *perturbational complexity index* (Casali et al., 2013) which measures the degree of consciousness even in comatose or locked-in state patients.

Given the complexity and importance of the topic, we deem it necessary to look also at some ethical implications at this point.

### 6.1. Ethical Implications

Being able to create artificial systems that are indistinguishable from natural conscious beings and thus are also potentially conscious raises ethical concerns. First and foremost, in the transformation from core consciousness to extended consciousness, the systems gain the ability to link new program routines. As such systems followings such a line of implementation need to be handled with care and should be experimented on in a contained environment. With the right choice of embodiment in a virtual machine or in a robotic body, one should be able to solve such problems.

Of course there are also other ethical concerns, the more we approach human-like behavior. A first set of robotic laws has been introduced in Asimov's novels (Clarke, 1993). Even Asimov considered the rules problematic as can be seen from the plot twists in his novels. Aside this, being able to follow the robotic laws requires the robot to understand the concepts of “humans,” “harm,” and “self.” Hence, such beings must be conscious. Therefore, tampering with their memories, emotions, and feelings is also problematic by itself. Being able to copy and reproduce the same body and mind does not lead to further simplification of the issue and implies the problem that we have to agree on ethics and standards of AI soon (Jobin et al., 2019).

## 7. Conclusion

In this article, we reviewed the state-of-the-art theories on consciousness in philosophy, neuroscience, AI, and machine learning. We find that the different disciplines need to interact to push research in this direction further. Interestingly, basic theories of consciousness can be implemented in computer programs. In particular, deep learning approaches are interesting as they offer the ability to train deep approximators that are not yet well-understood to construct mechanistic systems of complex neural and cognitive processes. We reviewed several machine learning architectures and related them to theories of strong reductionism and found that there are neural network architectures from which base consciousness could emerge. Yet, there is still a long way to form human-like extended consciousness.

## Data Availability Statement

The original contributions generated for the study are included in the article/supplementary material, further inquiries can be directed to the corresponding author/s.

## Author Contributions

All authors listed have made a substantial, direct and intellectual contribution to the work, and approved it for publication.

## Conflict of Interest

The authors declare that the research was conducted in the absence of any commercial or financial relationships that could be construed as a potential conflict of interest.

## Footnotes

^1^With this simple statement, Emil du Bois-Reymond concluded his talk on the limits of scientific knowledge about the relation of brain processes and subjective experience at the 45th annual meeting of German naturalists and physicians in 1872.

^2^Richard Feynman left these words on his blackboard in 1988 at the time of his death as a final message to the world.

^3^Note that of course, conscious brains have to be complex in order to implement a computing mechanism that is able to realize the process of model creation and selection accordingly. Thus, the concept of Occam's razor is by no means in contrast to this conviction. Rather, it describes the selection procedure of the models itself. According to Occam's razor, simple models should be preferred over more complex ones. The idea behind this approach is that a good model should, on the one hand be as complex as necessary, but on the other hand as simple as possible, i.e., not too complex, e.g., containing too many free parameters, that are not required to capture the phenomenon under consideration.

^4^In computer science, the concept of compression, or compressibility respectively, does not imply simplicity of the input or the model architectures. On the contrary, deriving more and more compressed representations from the input, requires highly complex processes for which in turn complex architectures are crucial. For instance, a random sequence of digits is incompressible but not complex. On the other hand, the infinitely many digits of π can be compressed to algorithms, i.e., representations, of finite length that (re-)produce the digit sequence of π with arbitrary precision. Thus, such an algorithm of finite length is both an extremely compressed representation of π, and highly complex.

^5^Depending on the specific task and input data, these connections are adjusted according to a certain learning algorithm (Bishop, 2006), e.g., supervised with error backpropagation (Chauvin and Rumelhart, 1995), unsupervised with, e.g., contrastive divergence (Carreira-Perpinan and Hinton, 2005) or through reinforcement learning (Sutton and Barto, 2018).

^6^These connections are hard-wired, i.e., not adjusted during training, and provide a copy of the current module's internal state to the same module. Thus, in each subsequent processing step, the module receives the current input together with its own previous internal state. This design principle enables processing of temporal structured data. So called recurrent neural networks (Tsoi and Back, 1997), like e.g., long short-term memories (LSTMs) (Hochreiter and Schmidhuber, 1997) have proven to do extremely well in speech and language processing, for instance.

^7^Training losses provide a reference during learning to adjust weights in trainable connections. In supervised learning (Bishop, 2006), the difference between actual and expected output, i.e., the error, serves as training loss (Chauvin and Rumelhart, 1995). In contrast, in reinforcement learning (Sutton and Barto, 2018), rewards indicating the appropriateness of a certain action or output are provided as feed-back.

^8^In principle, all presented models could be implemented, using state-of-the-art machine learning libraries such as Keras (Chollet, 2015), Tensorflow (Abadi et al., 2016), or PyTorch (Paszke et al., 2019). Of course, a lot of experiments are still necessary and some of the presented models are still difficult to train. However, some work toward this direction has already been done. For instance, some neural network based AI models capable of playing classic Atari games at human level do already incorporate world models of the games' environments (Kaiser et al., 2019), i.e., make use of so called model-based reinforcement learning (Sutton and Barto, 2018).
